# Effective Interventions in Obesity: Current Evidence and Concepts

**DOI:** 10.3390/nu15112511

**Published:** 2023-05-29

**Authors:** Karolina Szewczyk-Golec, Iga Hołyńska-Iwan

**Affiliations:** 1 Department of Medical Biology and Biochemistry, Faculty of Medicine, Ludwik Rydygier Collegium Medicum in Bydgoszcz, Nicolaus Copernicus University in Toruń, 85-092 Bydgoszcz, Poland; 2 Department of Pathobiochemistry and Clinical Chemistry, Faculty of Pharmacy, Ludwik Rydygier Collegium Medicum in Bydgoszcz, Nicolaus Copernicus University in Toruń, 85-094 Bydgoszcz, Poland; igaholynska@cm.umk.pl

This Special Issue, entitled “Specialized Diet, Obesity and Associated Metabolic Disorders” in the section “Clinical Nutrition” of Nutrients, addresses the metabolic changes that occur in humans as a result of the so-called western lifestyle. Overweight/obesity, metabolic syndrome and diabetes cause a number of metabolic changes to disrupt the homeostasis of the body, which, under certain conditions, may be reversible [1,2,3]. We have prepared for you a selection of original articles and reviews devoted to the assessment of the parameters of the oxidative–antioxidant balance, lipid and carbohydrate metabolism and the function of hormones during the use of various types of diets and lifestyle interventions supporting patients in the course of diseases related to overweight/obesity and metabolic syndrome.

One of the important directions of research is the evaluation of the impact of diet on changes in metabolism in patients with metabolic syndrome and comorbidities. Ferraz-Bannitz et al. [2] in a prospective, randomized controlled dietary study under constant nutritional and medical supervision showed that dietary protein-restricted intake can support the metabolism of patients with metabolic syndrome similarly to caloric restriction. Both the isocaloric restriction of proteins and the caloric restriction for 27 days had a positive effect on selected laboratory parameters, which were tested, among other things, in the material obtained by adipose tissue biopsy. Both types of diets contributed to weight loss, lowered blood glucose, lipid levels, and blood pressure, and improved insulin sensitivity. Thus, protein restriction, being more attractive and less drastic than caloric restriction, might be recommended as a complementary therapy in metabolic syndrome.

Interestingly, Lee et al. [3] showed that a 4-week use of a home meal replacement that was higher in protein and fat, lower in carbohydrates, and lower in the ω6FA/ω3FA ratio than a regular diet, might contribute to a reduction in body mass index (BMI), body weight, and parameters of the metabolic syndrome in adult patients with obesity. In turn, Świątkiewicz et al. [4] evaluated the impact of intensive rehabilitation and a plant-based diet on metabolism in patients with cardiovascular diseases. In a group of 314 patients, they showed that through supported lifestyle modifications, cardiovascular complications can be easily prevented and the risk of metabolic syndrome can be reduced. A particularly negative impact of intestinal microbiota dysfunction on the functioning of the vascular endothelial cells was described by Choroszy et al. [5] in patients with coronary artery disease. Research results indicate that damage to the microbiome caused by a high-fat diet and smoking results in an increase in the level of bacterial metabolites in the blood of patients with cardiovascular diseases, which strongly stimulates endothelial inflammation and atherosclerotic processes, promoting the development of cardiovascular complications. In a review of research on intermittent energy-restricted diets, Stanek et al. [6] presented the benefits of using diets consisting of intermittent pauses in eating in the regulation of glucose and lipid metabolism, as well as blood pressure. The authors emphasized that these types of diets are as efficient as diets with continuous energy restriction. Moreover, intermittent energy restriction could be recommended to patients for whom maintaining a constant nutritional regime is particularly inconvenient and difficult to accept.

In a porcine model of non-alcoholic fatty liver disease, the effect of “western diets”, i.e., high-fructose and high-fat diets, with or without probiotics, on muscle oxidative stress and muscle remodeling, was evaluated [7]. Rapidly increased oxidative stress, inflammatory state and muscle remodeling were found in young animals due to the use of an improper diet. However, the use of probiotics significantly improved the measured parameters. In turn, Poloczek et al. [8] showed that the use of a proper diet after bariatric duodenojejunal omega switch surgery is crucial for the normalization of hepatokines, such as fetuine-6, pentraxin-3, and growth differentiation factor-15, in obese rats.

Of particular interest is the possibility of improving parameters related to metabolic diseases and weakening the effect of an incorrect diet by using bioactive substances. The oral administration of punicalagin, a polyphenol from pomegranate, was proven to have antioxidant and lipid-regulating effects, despite the use of high-fat diets in mice with diabetic liver failure [9]. It was found that, in the liver, punicalagin stimulates, among other things, the expression of catalase and manganese superoxide dismutase and increases the mitochondrial membrane potential. In another study on a model of the intestine, it was shown that the use of *Cornus mas* fruit extract inhibits the activity of alpha-amylase and lipase, which may lead to a decrease in the absorption of glucose and lipids, and at least partly explains the use of cornelian cherries in diabetes and obesity [10]. On the other hand, the oral administration of soybean-derived peptides positively regulated hyperlipidemia and lipid metabolism in the liver by downregulating the expression of cytochrome p450 family 7 members in mice [11]. Interestingly, an analysis of the results of basic preclinical studies conducted by Canaan et al. [12] showed that the use of yeast beta-glucans had little effect on body mass reduction. Particularly noteworthy is the fact that the authors analyzed only registered studies on rat models, which significantly increases the credibility of the conclusions drawn.

The problem of obesity and its complications is particularly difficult in the case of patients suffering from diseases conducive to its development, such as polycystic ovary syndrome. For this reason, the study conducted on girls aged 14–18 described by Mizigier et al. [13] seems to be of primary importance. Healthy eating habits and physical activity were shown to normalize the levels of thyroid hormones and androgens in patients with polycystic ovary syndrome, thus reducing the incidence of disease symptoms. Dubey et al. [14] reviewed the current literature data on the effect of vitamin, mineral and probiotic supplementation on oxidative stress, lipid profile and carbohydrate metabolism in women with polycystic ovary syndrome. The main conclusion of the studies discussed in the review is that lifestyle changes and increased physical activity could effectively reduce oxidative stress and regulate the lipid profile and glucose concentration, which translates into a lower risk of metabolic diseases, in particular cardiovascular complications.

A study helpful in understanding the processes underlying the development of metabolic syndrome was described by Gromova et al. [15]. In the experiment, they studied young rats that developed diabetes. It was shown that the younger the individual exposed to a high sugar supply, the more glucose transporters are present in the enterocyte plasma membrane located in the enterocyte brush border, which significantly affects the intestinal microenvironment. In turn, Stanek et al. [16] collected and systematized scientific data on perivascular adipose tissue. Research on adipose tissue is extremely important for understanding the pathomechanisms of atherosclerosis and hypertension. The authors demonstrated that obesity leads to the development of oxidative stress in adipocytes surrounding the vessels, which initiates inflammation and leads to the development of atherosclerosis, hypertension and metabolic syndrome.

When you enter the keyword “metabolic syndrome” in PubMed, a search engine that provides access to databases of publications in the field of life sciences and biomedical issues, nearly 60,000 scientific articles are displayed. Research conducted on humans, as well as on animal and *in vitro* models, allows for a better understanding of the processes involved in the development of obesity and its complications, as well as to answer the question to what extent lifestyle changes, physical activity, various dietary interventions and supplementation with bioactive substances could restore hormonal balance, regulate metabolism and reduce the risk of metabolic diseases. As overweight/obesity and metabolic syndrome constitute a significant health, social and economic problem for an increasing number of countries in the world, and they are the cause of the majority of deaths in developed countries, research in this area should be a priority. The presented Special Issue is an interesting selection of up-to-date research results on this subject.
